# Trick or Treating in Forensics—The Challenge of the Saliva Microbiome: A Narrative Review

**DOI:** 10.3390/microorganisms8101501

**Published:** 2020-09-29

**Authors:** Gabriella D’Angiolella, Pamela Tozzo, Sarah Gino, Luciana Caenazzo

**Affiliations:** 1 Department of Cardiac, Thoracic, Vascular Sciences and Public Health, University of Padova, 35121 Padova, Italy; dangiolellagabriella@gmail.com; 2 Department of Molecular Medicine, Laboratory of Forensic Genetics, University of Padova, Via Falloppio 50, 35121 Padova, Italy; luciana.caenazzo@unipd.it; 3 Department of Health Sciences, University of Piemonte Orientale, 28100 Novara, Italy; sarah.gino@uniupo.it

**Keywords:** saliva microbiome, stability of saliva microbiome, diversity of saliva microbiome, forensic sciences, human identification

## Abstract

The oral microbiome harbours microbial community signatures that differ among individuals, highlighting that it could be highly individualizing and potentially unique to each individual. Therefore, the oral microbial traces collected in crime scenes could produce investigative leads. This narrative review will describe the current state-of-the-art of how the salivary microbiome could be exploited as a genetic signature to make inferences in the forensic field. This review has been performed following the Preferred Reporting Items for Systematic Reviews and Meta-Analyses (PRISMA) Guidelines. Even if further studies are needed to relate the variation in the oral microbiome to specific factors, in order to understand how the salivary microbiome is influenced by an individual’s lifestyle, by reviewing the studies published so far, it is clear that the oral microbial analysis could become a useful forensic tool. Even if promising, caution is required in interpreting the results and an effort to direct research towards studies that fill the current knowledge gaps is certainly useful.

## 1. Introduction

Since bacteria were described in 1683 by Antonie van Leeuwenhoek in his letter to the Royal Society as “an unbelievably great company of living animalcules” [1], we have learned how to culture, isolate, and characterize hundreds of microbial species. The advent of culture-independent techniques has also made it possible to obtain information on (so far) unculturable microbes [2].

Nowadays, it is known that the human body hosts a great number of microbial cells [3]. The microbiome, which has been defined as the total genetic content of microbes inhabiting our bodies [4], differs massively in species composition and abundance at different body sites, with distinct microbial community signatures that appear to be specific to particular areas of the body [3].

The oral cavity is one of the major gateways to the human body and harbours hundreds of species of bacteria in addition to viruses and fungi. The oral cavity offers numerous different microbial habitats, with shedding (mucosa) and solid surfaces (teeth or dentures). Each different habitat, such as dental surfaces; the gingival crevice or sulcus; the tongue, with its papillae and crypts; and different keratinized (gingival and hard palate) and non-keratinized (cheeks, lips, soft palate) mucosal surfaces, contains specific ecological niches that select for distinct microbes [5]. It has been hypothesized that one millilitre of human saliva from a healthy adult yields around 100 million microbial cells. At the normal salivary flow rate, which is 750 mL/day, about 8 × 1010 bacteria are shed from the oral surfaces every 24 h [6].

These microorganisms constitute the human oral microbiome, which comprises more than 2000 bacterial taxa, including a large number of opportunistic pathogens. Even if oral microbiome represents the second most diverse microbial community following stool, playing a crucial role in determining human health and diseases, it has been understudied compared with the gut microbiome. With the advent of DNA sequencing advances, much progress has been made in understanding the complexity of the oral microbiome [2]. The most important efforts are focused on recognizing the potential connections between oral microbial communities and a wide range of oral [7] and systemic pathological conditions [8], such as sepsis/endocarditis, type-2 diabetes, and cardiovascular diseases, as well as their established risk factors, such as obesity, chronic kidney disease, Alzheimer’s disease, rheumatoid arthritis, and head and neck cancer [7,8,9,10,11,12].

Notably, the oral microbiome harbours microbial community signatures that differ among individuals, highlighting that it could be highly individualizing and potentially unique to each individual [13]. Therefore, the oral microbial traces collected at crime scenes could produce investigative leads in criminal and civil cases.

This narrative review describes the current state-of-the-art of how the salivary microbiome could be exploited as an individual signature to make inferences about characteristics and qualities of the likely offender or victim in forensic crime investigations.

## 2. Materials and Methods

This review was performed following the Preferred Reporting Items for Systematic Reviews and Meta-Analyses (PRISMA) Guidelines [14].

The articles identified in the present review were selected from PubMed and Scopus databases; for the search strategy, we decided to use the following keywords: “(microbiota or microbiome) AND (saliva OR oral)” and “(microbiota OR microbiome) AND forensic”. We used the keywords isolated or combined. We searched for multiple studies among the reference lists of the selected papers and systematic reviews. In this way, we identified a total of 1579 works on PubMed and Scopus databases.

Two of the reviewers (P.T. and G.D.) carried out the initial search of the papers. They used the search protocol described above to identify literature. In the case of disagreements, the consensus of the research supervisors (L.C. and S.G.) was asked. The researchers used the following research order: titles were screened first and then abstracts and full papers. A paper was considered potentially relevant and its full text reviewed if, following discussion between the two independent reviewers, it could not be unequivocally excluded on the basis of its title and abstract. The full text of all papers not excluded on the basis of abstract or title was evaluated.

Duplicates were removed and a total of 628 works were screened on the basis of the inclusion criteria: (1) abstract and full text in English language; (2) titles and/or abstracts suggested a personal identification performed by salivary microbiome analysis; and (3) titles and/or abstracts suggested the analysis of salivary microbiome association with age, ethnicity, and smoking habits. A total of 189 article abstracts were included during this phase of abstract screening, after which 59 articles were examined in their full-text form for eligibility.

In the end, after full-text examination, we selected 19 experimental studies published between 2005 and 2020 for qualitative synthesis.

To estimate the potential biases that were most relevant for the study, we used the Cochrane tool for assessing the risk of bias in randomized trials (RoB 2 tool) and the risk of bias in non-randomized studies of interventions (ROBINS-I). All articles included are considered to be of low risk of bias.

**Figure 1:**
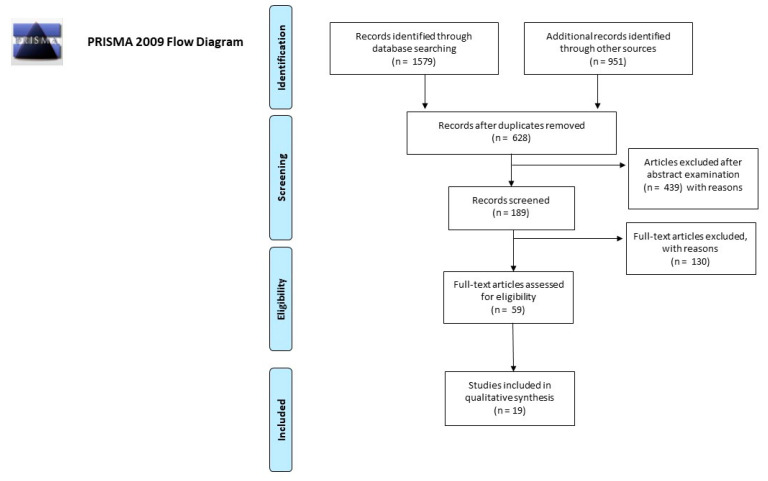
Preferred Reporting Items for Systematic Reviews and Meta-Analyses (PRISMA) 2009 flow diagram.

The number of articles excluded or included was registered and reported in a PRISMA flowchart (Figure 1).

## 3. Results

In order to make the results obtained in the current review more comprehensible to the reader, we have summarized the selected studies by grouping them into the following four categories: (a) personal identification for forensic purposes; (b) oral microbiome in age prediction; (c) signature of ethnicity in oral microbiome; and (d) oral microbiome as indicator of personal habits: smoking. 

The potentials and limitations of saliva microbiome for forensic purposes are summarized in Table 1.

### 3.1. Personal Identification for Forensic Purposes

Before tackling the topic of forensic personal identification, it is important to consider the published studies to date related to salivary microbiome changes over time. Indeed, variation in human oral microbial composition could compromise the forensically fundamental purpose to link crime scene traces to the subject(s) who left them. The microbial composition has to remain the same both in collected traces and in a suspect’s oral cavity to be useful for forensic purposes: temporal changes in microbial crime scene traces or an individual’s salivary microbial communities might result in a failure of the match with the exclusion of the perpetrator from the investigation of the crime.

The first studies on the characterization of salivary microbiome date back to the early 2000s and rely on genetic fingerprinting techniques, including denaturing gradient gel electrophoresis (DGGE), which was widely employed for characterization and profiling of bacterial communities because it provides qualitative and semi-quantitative information about both mixed microbial populations and temporal changes in a community’s composition. As the DGGE enables the simultaneous analysis of multiple samples, it enables the easier comparison of microbial composition in different samples. Furthermore, the amplified bands in DGGE can be excised from the gel and can be sequenced in order to obtain a taxonomic identification.

In 2005, Rasiah et al. [15] explored the composition and stability of human saliva and dental plaque microcosm biofilm grown in the multi-plaque artificial mouth (MAM). In vitro, the MAM system allows the growth of plaque microcosm biofilm, which is a substance that resembles natural plaque. Rasiah et al. collected saliva from 10 volunteers, who did not practice any oral hygiene for at least 24 h, and who chewed a chicle gum prior to saliva collection. Furthermore, a standard donor was chosen from among the 10 volunteers. His saliva was collected over a long period, lasting 7 years, from 1998 to 2004. The authors conducted multipurpose research. First, they used DGGE in order to evaluate temporal changes in bacterial communities from the standard donor over a 7-year period. The visual inspection of DGGE patterns revealed that the variation between samples was principally attributable to differences in band intensity, and not to the appearance of new bands. Therefore, Rasiah et al. inferred that, although there were some transient changes, the salivary bacterial composition was relatively stable over time. The authors also tried to identify whether the oral microbiome is host-specific, by comparing the saliva bacterial patterns from the 10 different volunteers. The results showed a pronounced variation in band patterns between individuals. This inter-individual variation was also greater than the variation observed in the time series from a single individual. Finally, the authors investigated whether the bacterial communities changed from the 10 individual saliva samples into the corresponding mature plaque microcosm biofilms developed in the MAM system for 24 days. The results showed that, during plaque microcosm development, changes in bacterial composition occurred. In fact, the authors detected a 20% reduction in similarity between the 10 plaque profiles compared with the overall similarity between the original 10 saliva samples. In addition, dominant species yielded in saliva were different to those developed in plaque microcosms, suggesting that the selective pressures imposed by the environment could cause changes in oral bacterial communities.

In 2009, Costello et al. studied the long-term stability of the oral bacterial population over time by analyzing 27 body sites in healthy adult volunteers, including the oral cavity, four times on day 0, day 1, day 90, and day 91 [16]. The differences in the overall bacterial community composition were assessed using Unifrac distances. Costello et al. highlighted that body habitats differed in the degree of temporal variation. The oral microbiome showed a marked degree of spatial and temporal stability, especially compared with microbial communities in other body sites. Even if intrapersonal differences (over time) were smaller than interpersonal differences (on each day) within all habitats examined, the oral microbiome showed smaller temporal diversity than the gut and the skin ones. The authors concluded that the size of the set of phylotypes shared among all individuals, which can be defined as the community “core”, will depend on the body habitat examined, and is likely to be larger in the oral cavity than in other habitats such as the gut or skin.

Lazarevic et al. [17], in 2010, compared saliva samples from five different individuals in terms of the phylogeny of their microbial communities within a 29-day period. The authors, by performing the salivary bacterial community comparisons using UniFrac distances, noticed that the samples collected from the same individual were clustered. In particular, the results showed that the salivary microbial community appeared to be stable over at least 5 days. It was also possible (for three subjects) to obtain a subject-specific grouping even analysing samples collected at more distant time points (15–29 days). Lazarevic et al. demonstrated a relative stability of the salivary microbiome, with samples collected at closer sampling times, that did not appear to be more similar than samples collected across longer time intervals.

As shown by Zhu et al. [18] in a study conducted in 2012, the temporal stability of oral microbial composition can also be influenced by specific treatment with oral dental prostheses, such as removable partial dentures (RPDs). The authors enrolled 10 volunteers with Kennedy I dentition defect and a similar RPD coverage range. A control group composed of 10 healthy individuals without any prostheses was also chosen. During a temporal interval of 6 months, various samples were periodically collected, at three time-points: just before wearing RPDs, as well as at 1 month and 6 months after the treatment. Zhu et al. evaluated bacterial diversity by using DGGE. In samples collected pre- and post-wearing of RPDs, the authors noticed a significant difference in the number of amplicons, demonstrating that the oral microbial ecosystem had been re-established by the treatment. Interestingly, in the control group, at different time points over a 6-month temporal interval, the predominant bacterial communities were stable.

The first study that evaluated the possibility of differentiating the oral microbiome at an individual level was published in 2012 by Stahringer et al. [19], who investigated the variability of the oral microbiome on twins and siblings. The authors studied a large human cohort, made up of 107 individuals between the ages of 8 and 26, to examine their microbial composition and to determine how it was influenced by human genotype, gender, age, and weight class. In order to evaluate if the composition of human microbiome was inheritable, Stahringer et al. enrolled among the participants 27 monozygotic and 18 dizygotic pairs of twins, 8 unrelated pairs of adopted siblings, and 1 unrelated individual from the same cohort. The authors collected a total of 264 saliva samples. According to what has been previously described, the results showed that the main bacterial phyla in saliva were *Firmicutes*, *Proteobacteria*, *Bacteroidetes*, *Actinobacteria*, and *Fusobacteria*. Interestingly, it was possible to define a core salivary microbiome at the genus level, because, in >95% of all samples, the following eight genera were observed: *Streptococcus*, *Veillonella*, *Gemella*, *Granulicatella*, *Neisseria*, *Prevotella*, *Rothia*, and *Fusobacterium*, and an additional thirteen genera were detected in >50% of the samples. Even if monozygotic twins share 100% of their alleles and dizygotic ones only about 50% of their alleles, using the unweighted Unifrac distance, the authors observed that the difference between the microbial communities of each pair of twins was not statistically different, with only a slight trend toward greater similarity among cohabiting monozygotic pairs than dizygotic ones, suggesting a small genetic influence on microbiome composition. Eighty-two individuals were sampled by Stahringer et al. more than once (198 saliva samples), at up to three time-points for ten years spanning adolescence in order to detect temporal changes in oral microbial composition. The authors highlighted that, after 5 years, the oral microbiome of an individual resembles itself more closely than that of the population, but after 10 years, the self-similarity was no longer statistically significant. Furthermore, the similarity across the twin pairs appeared to decrease between the ages of 17 and 22, when 84% of twin pairs stopped cohabiting. Stahringer et al. concluded that the environment plays an important role in the overall composition of the oral microbiome, with a remarkable long-term stability of the oral microbiome over at least 5 years.

In 2016, Leake et al. [13] analysed the intra and inter-individual variation of the salivary microbiome of two healthy subjects to demonstrate the potential of NGS (Next Generation Sequencing) analysis of the salivary microbiota for forensic identification. The authors collected saliva samples from two healthy adult individuals who were asked to spit into a sterile tube at four time points; t = 0 and t = 30 days and one year later at t = 0 and t = 30. These volunteers brushed their teeth in the morning and did not eat or drink one hour before sampling. In this study, two different targets, namely 16S rRNA and rpoB, were analysed. The 16S rRNA gene has been widely used for phylogenetic studies [20,21]. In addition, in order to investigate the biodiversity of *streptococci* and other bacteria, two different pairs of primer targeting were used, namely rpoB1 and rpoB2. For 16S rRNA, primers were designed to amplify the V5 region and, for rpoB, two sets of primers covered the V1 region.

For both rpoB1 and 16S rRNA, *Firmicutes* was the most common phyla, constituting over 90% and 70% of the population, respectively. For rpoB2, the population was composed of over 90% *Actinobacteria*. This large difference in taxa found by each rpoB primer pair was attributable to the fact that these primers were designed to amplify different taxa, thus demonstrating the benefit of targeting more than one region of the same target gene. Additional rpoB allowed for the analysis of certain genera down to the species and even strain level. In particular, by analysing 16S rRNA, Streptococcus could be characterised at the genus level and occasionally the species level (nine different OTUs – Operational Taxonomic Units); by analysing rpoB, it could be detected to the species/strain level (53 different OTUs), obtaining a deeper characterization.

However, Leake et al. found that the most common phyla in saliva were *Firmicutes*, *Proteobacteria*, *Actinobacteria*, *Bacteroidetes*, and *Fusobacteria*, as demonstrated by previous studies. They also showed that, by combining three targets, it was possible to observe a genus-level core microbiome of 58 genera. This high number of genera covered about 95% of the population of each individual, implying that most differences came from the species/strain level. In order to provide a good separation between individuals with all targets, it was necessary to have a minimum number of sequences of about 100,000. Discrimination was not significantly increased by the addition of rpoB2. In fact, even though the best separation was achieved with sequences of all three target genes, when combining only 16S rRNA and rpoB1, it was still possible to achieve a very good separation.

Leake et al., using a combination of a highly discriminative gene (rpoB) with the 16S rRNA target generally used for PCR-based metagenomics, investigated a technique that could be used for human identification, especially when current methods, based on human DNA typing, cannot be utilized. The authors concluded that the Illumina high-throughput sequencing of the salivary microbiome could be used to identify saliva samples from two different individuals.

In 2019, Wang et al. [22], taking into account that, when there are a lot of samples, the cost of next-generation sequencing rises, making it difficult to conduct a study, searched for a rapid and low-cost method to be applied before the sequencing. The authors designed a general primer pair targeting the 16S rRNA V4 region of bacteria. They collected a total of 10 samples, from 5 healthy volunteers (two males and three females), who have not taken antibiotics in the past three months and who were asked not to eat and drink at least one hour before sampling. Each volunteer contributed to the study with a saliva sample and an oral swab sample. At the end of the PCR protocol, Wang et al. performed a high-resolution melting analysis, which revealed the presence of distinct microbial communities and showed that the amplicon melting curve profiles were different among the five saliva samples, except for two samples, which provided a similar melting curve profile. These two samples came from volunteers who shared the same environment and who followed a similar diet. Furthermore, the authors observed that the saliva samples and oral swab samples from the same individual matched well, except in one case. This discrepancy was explained by the authors considering that the saliva sample could yield throat microbiome, in addition to oral microbiome. Wang et al. concluded that the human oral microbiome should be studied with a more accurate method, such as sequencing, to more deeply analyse the discrepancy in different samples because it has the characteristics to become a marker in personal identification.

In a study published in 2020, Sundström et al. [23] investigated, using 16s rRNA gene amplicon sequencing, the relatedness of salivary sample microbiome collected from members of the same family. In order to do so, the authors enrolled, as study subjects, two volunteer families. The first one was a family of three generations, composed of 10 adults, and the second one was an unrelated family of two generations, composed of 4 adults.

Saliva samples were collected by spitting into sterile plastic vials, after not having eaten and drunk for at least 2 h. After DNA extraction, all samples were amplified using primers targeting the V3–V4 regions on 16s rRNA gene. The authors calculated beta diversity using unweighted UniFrac method. Beta diversity metrics describe the degree to which samples differ from one another. Furthermore, the authors performed an Adonis test to study the differences in microbiome composition between the two unrelated families. Unfortunately, two subjects were excluded from the study. The first one because he was the only smoker; the second one because the saliva sample was mixed with blood and the sequencing results showed a dominance for pathogenic bacteria, associated with periodontitis.

The authors performed differential abundance analysis with two databases (SILVA and Human Oral Microbiome Database (HOMD)). Nevertheless, they chose to present results with SILVA, which is the older of the two databases and the one that has been considered as the gold standard for a long time. On the other hand, HOMD is a relatively new database and is smaller than SILVA. In fact, even if the human oral microbiome yields about 700 species, only 400 bacterial species are listed in HOLD.

According to SILVA, the major phyla were *Firmicutes*, *Bacteroidetes*, *Proteobacteria*, *Fusobacteria,* and *Actinobacteria* (38% of the total identified phyla). The most common genera were *Streptococcus* spp., *Veillonella* spp., *Prevotella* spp., *Neisseria* spp., and *Leptotrichia* spp. (3.7% of the total identified genera). The five most abundant taxa were unclassified *Synergistaceae*, *Atopobium* spp., *Human oral bacterium BD1-5*, *Lactobacillus* spp., and *Butyrivibrio* spp. The above-mentioned differences between the two databases resulted in the fact that the unclassified *Synergistaceae*, which was the most abundant taxa according to SILVA, was not recognized by HOMD.

The results showed that 13% of the variance between individuals’ bacterial communities could be explained by family ties (the R2 value obtained from the Adonis test was of 0.13; *p* = 0.001). The authors observed that, compared with fathers, mothers shared more OTUs with adult children. Sundström et al. explained this result considering that the mother’s microbiome highly contributes to the son’s colonization during childhood, from the birth canal during labor, from breast milk, and from the strong and close physical contact between the mother and infant in infancy. However, this similarity appeared to become weaker (but did not disappear) as the time passes by and the sons grow up, showing that the mother still influences her sons’ oral microbiome in adulthood.

In addition, the authors noticed that the highest resemblance was observed between parents and younger adult children, who still live with them, suggesting the great influence of environmental factors, such as cohabitation.

### 3.2. Oral Microbiome in Age Prediction

Predicting the lifetime age of an unknown perpetrator at the time of trace deposition could greatly aid police in focusing their investigation in order to find unknown trace donors. The hypothesis that this type of information can provide through the analysis of the salivary microbiome has been proposed in the literature, but only two studies are available with interesting, but obviously not definitive results.

Huang et al. [24] investigated the possibility of predicting age in adults using random forest (RF) regression on data combined from multiple publicly available studies. In particular, the authors used RF to regress relative abundances of amplicon sequence variants (ASVs) against the subjects’ chronological ages. According to their results, even if the skin microbiome provides the best prediction of age, the oral microbiome analysis, which was performed on 2500 saliva samples, could also pinpoint a subject’s age to within 5 years on average. Huang et al. identified that some microbial markers could contribute to the age-prediction model, by decreasing in abundance as the host ages, in both females and males. In particular, these markers in the oral microbiota were ASVs belonging to *Lactobacillales*, *Gemellaceae*, *Bacteroides*, and *Fusobacterium*.

Murugesan et al. [25] analysed the relative abundance of the salivary microbiome in 997 volunteers, revealing that the elderly ones (people older than 65 years old) showed a lower bacterial richness and diversity as compared with their adult counterparts, aged under 65 years old.

### 3.3. Signature of Ethnicity in Oral Microbiome

In 2009, Nasidze et al. [26] sequenced a part of the 16S rRNA from 120 individuals. Volunteers were from 12 locations worldwide (10 for each location). The authors demonstrated a significant association between variation in the saliva microbiome and the distance of each location from the equator. Starting from these results, Li et al. in 2014 [27] explored the variation in the composition of the oral microbiome by analysing the salivary microbiome diversity of three human groups who live under different climates and geographic regions. In particular, the authors included in their study native Alaskans, Germans, and Africans. They enrolled 76 individuals from four native Alaskan communities, and 10 individuals living in or nearby Leipzig, in Germany. In order to obtain a more informative comparison, they also included in their study published data previously generated with similar methods for three groups from Africa (a total of 66 individuals from three different populations, located in the Democratic Republic of Congo, Sierra Leone, and Uganda). Volunteers, aged from 20 to 40 years, were asked to spit about 2 mL of saliva into tubes containing 2 mL of lysis buffer. The authors did not investigate the oral health of the donors, although they did not suffer from full-blown oral diseases. Furthermore, Li et al. did not study the correlation between demographic characteristics (age and gender) and oral microbiome variation. The study, in fact, focused only on the effect caused by different geographic and climatic areas on salivary microbiome composition.

Li et al. analysed differences among populations at both the genus and OTU level. Regarding the four Alaskan groups, even if they are localized in different regions, with Atqasuk and Nuiqsut sited in the inland, and Barrow and Wainwright along the coast, there were no significant differences. On the contrary, the geographic location and the consequently different diets seemed to play an important role in influencing the microbiome composition in the African groups.

In addition, the authors observed that the populations from the northern continents were of comparable similarity, with the German group showing a high similarity with the Alaskan ones at the genus level, while keeping distinct differences at the OTU level.

In order to further evaluate the differences among the salivary microbiomes of the three geographic regions, the authors calculated alpha diversity (within individual diversity) using the Shannon–Weaver Index, and beta diversity (inter-individual diversity) using the Sørensen Index. The highest alpha diversity was shown by Germans, while the lowest was shown by the African group. The authors explained the differences in alpha diversity in the German group, taking into account the wider variety of food, with a great amount of different carbohydrates, that characterizes the German diet compared with that of native Alaskans or Africans. The more complex availability of nutritive substrates could allow for the colonization of the oral environment by a higher number of bacterial communities. Furthermore, the higher population density could facilitate the spread of bacteria among people.

Regarding beta diversity, it was higher in the African group where, despite the relatively low diversity within individuals, the large geographical area makes the group more geographically dispersed, with an increase in the overall diversity of observed bacteria.

In conclusion, the study conducted by Li et al. demonstrated how human populations from different geographic and climatic areas exhibit differences in their salivary microbiome, corroborating what was previously shown by Nasidze et al. [26] about the association between Unifrac distances and the geographical distance of analysed individuals from the equator.

A particular signature of ethnicity in oral microbiome was also underlined by Sarkar et al. [28] in 2017, when they analysed microbiome diversity in saliva samples collected from 92 healthy volunteers, coming from eight different geographical locations in India. These different locations represented three geographic regions in India. Even if the investigated Indian population showed an important bacterial richness, with 165 bacterial genera and 785 OTUs, the authors highlighted an extensive sharing of OTUs (37 OTUs that could be assigned to 12 bacterial genera), representing a putative core microbiome for the Indian population. Furthermore, on the basis of both genera distribution and Unifrac metrics on OTU abundance, Sarkar et al. observed small, but significant correlations in the abundance of bacterial genera in samples belonging to volunteers from the same geographic region, suggesting that geographical proximity could increase sharing of salivary microbiome. In their study, Sarkar et al. adopted a deep-sequencing approach that allowed for the detection of extremely rare microbial species, with the detection of 54 OTUs that were not previously reported in the Human Oral Microbiome Database (HOMD). The authors assessed in this study that the latitude, as a function of distance from the equator, could also explain a significant fraction of the variance in the oral microbiome.

In 2020, Murugesan et al. [25] determined the salivary bacterial composition of the Qatari population by analysing 997 saliva samples collected from a group of adult (aged at least 18 years) volunteers composed of 442 males and 555 females. Furthermore, the authors aimed to assess the role played by factors such as gender, age (as previously shown), oral health, smoking, and some dietary habits in the salivary microbiome composition. Therefore, they took into account the demographic and clinical characteristics of these volunteers, including oral hygiene practices. The authors confirmed the hypothesis of a population-based variability in the salivary microbial profiles by comparing the salivary microbiome composition in the samples collected from Qataris with the microbial profiles from populations included in NCBI/SRA (National Center for Biotechnology Information/ Sequence Read Archive) bioprojects.

### 3.4. Oral Microbiome as Indicator of Personal Habits: Smoking

As the changes of the oral microbiome induced by cigarette smoking could be associated with several systemic diseases, the relationship between cigarette smoking and the oral microbiome has been widely studied so far. Smoking alters the composition of the oral microbiome by decreasing the commensal microbial population and increasing the pathogenic one, as highlighted in 2016 by Kato et al. [29], who, by analysing mouth rinse samples, pointed out that, in current smokers, Neisseria was less abundant, while bacterial members of the *Veillonellaceae* family were more abundant.

In a study conducted by Yu et al. in 2017 [30] on 23 current smokers and 20 never-smokers, by investigating the effect of cigarette smoking on different sites of the oral cavity, the authors noticed that the microbial diversity and composition were significantly different by smoking status only on the buccal mucosa, where the alpha diversity was lower in smokers than in non-smokers. Yu et al. did not highlight significant differences of microbiota across the other examined oral sites (nine samples per subject, including saliva) related to smoking. The fact that smoking causes oxygen deprivation, resulting in oral proliferation of anaerobic species, was demonstrated in 2019 by Wu et al. [31], who tried to assess the relationship between cigarette smoking and the oral microbiome by analysing oral wash samples from 1204 American adults. In particular, in order to evaluate if overall microbial composition differed between never, former, and current smokers, the authors analysed oral bio-specimens collected from participants in two distinct cohorts between 1993 and 2002. After controlling for age, sex, and data set, the authors noticed a significant difference in oral bacterial communities according to smoking status, with current smokers different from the non-current smokers group, made up of former and never-smokers. In particular, Wu et al. highlighted a depletion of the phylum *Proteobacteria* and an elevated relative abundance of the phyla *Firmicutes* and *Actinobacteria* among current smokers. Furthermore, by comparing the within- and between-group distances, the never and former smokers turned out to be more alike than the current smokers, who appeared to be a more heterogeneous group, suggesting that smoking-related changes are not permanent and the bacteria depleted by cigarette smoking could be restored after quitting smoking. Similar results were obtained by Yang et al. [32], who conducted a study on a large population of predominately low-income and African-American participants, including 592 current smokers, 477 former smokers, and 547 never-smokers. The authors, considering that the oral microbial composition of current smokers differed from former and never-smokers and that such differences were not observed between former smokers and never-smokers, concluded that the changes caused by the strong impact of smoking on oral microbial composition may be recovered after smoking cessation.

Similar results were obtained by Grine et al. [33], who collected saliva specimens from 90 different individuals (19 smokers and 71 non-smokers). These specimens were specifically investigated for the presence and diversity of Gram-positive bacteria using a routine culture and identification protocol. According to their results and concordantly with investigations conducted with culture-independent methods, tobacco smoking reduced the diversity of saliva microbial composition. In particular, in this experiment, Gram-positive bacterial species were reduced from 18 in non-smokers down to 7 in smokers. Because all the species cultured in smokers were also cultured in non-smokers, the authors hypothesized that smoking has some deleterious effects on the 12 species that have been specifically found in non-smokers, but have not been cultured in smokers, inhibiting their growth in smokers.

In 2019, Al-Zyoud et al. [34] included in their study 100 healthy subjects (57 males and 43 females), with 51 non-smokers and 49 smokers. The smokers used to smoke at least one cigarette per day. By targeting the 16 rRNA gene, the authors showed that smoking, regardless of gender, even with slight significant statistical variation between males and females in general, causes changes in salivary microbial composition, resulting in the possibility of classifying smokers and non-smokers at the genera level, by LEfSe (Linear Discriminant Analysis effect size), which is a biomarker discovery and explanation tool for high-dimensional metagenomic data. In particular, the results showed that six genera (*Streptococcus*, *Prevotella*, *Veillonella*, *Rothia*, *Neisseria*, and *Haemophilus*) were predominant, even if with different proportions, in all collected samples. Furthermore, the authors noticed that the salivary abundance of *Streptococcus*, *Prevotella*, and *Veillonella* was greater in smokers relative to non-smokers, at the expense of *Neisseria*, which was more abundant in non-smokers relative to smokers.

In the study published in 2020 by Murugesan et al. [25], and already discussed about ethnicity and age, the influence of smoking on the salivary microbiome composition was also studied. The authors, by classifying the participants into two groups, the smokers (264 subjects) and the non-smokers (733), showed that the salivary microbiome of the non-smokers was significantly more diverse as compared with the smokers, but the species richness was not significantly different between the two groups.

The results of the studies investigating the impact of cigarette smoking on the oral microbiome were inconsistent.

## 4. Discussion

Numerous major advances have been made in the scientific discipline of microbial forensics over the past decade. Most of the studies published so far have been focused on the analysis of the skin microbiome to connect people to an object or a place with important repercussions in the forensic field, shining a spotlight on the possibility of undertaking a microbial forensic investigation to identify the perpetrator of a crime [35]. The most important questions to be answered by forensic science are usually both “who committed the crime?” and “who did not commit the crime?”. It is known that crime scene evidence analysis can play a pivotal role in answering the above-mentioned questions.

As saliva is a bodily fluid that can be collected as evidence from a crime scene, we have summarized how information derived from salivary microbial analysis can associate or exclude individuals, victims, and/or suspects with/from a crime.

Whereas salivary microbiome shows considerable variation between subjects, the analysis of the time-dependent variation in salivary microbial composition has revealed a long-term stability or little changes within the same subject [13,15,16,17,18].

Regarding the potential of NGS analysis of the salivary microbiome for forensic identification, the microbiome profile of the saliva can be particularly helpful in those cases when the aggressor’s DNA is mixed with the victim’s DNA, such as in sexual assault cases, especially if the perpetrator’s DNA traces are barely detectable and insufficient to determine a complete and reliable genetic profile [13]. Furthermore, salivary microbial analysis can play a fundamental role in distinguishing monozygotic twins, who share 100% of their genes [19]. Current forensic DNA profiling is completely a comparative analysis, with the purpose of matching DNA profiles from crime scene traces with that of known suspects, such as those included in forensic DNA databases because of previously committed crimes. The principle limitation of this kind of analysis is that perpetrators whose DNA profiles are unknown to the investigators, both because they are not listed as likely offenders and because their profiles are not included in such databases, cannot be identified. For these reasons, we consider that the real forensic usefulness of oral microbial analysis might consist of adding a new qualitative dimension to criminal investigations. Microbial analysis can overcome previously mentioned limitations of the current use of DNA in human forensics. It might allow further characterization of unknown perpetrators, in order to guide police investigations towards the most likely group of potential suspects, when it is not possible to identify direct suspect(s) by conventional means of investigations, especially in cases where the police have little or no knowledge of the identity of the trace donor and how to find him/her. However, certain limits must be kept in mind to avoid overestimating the real possibilities of this tool in forensics. Some of these limitations, for example, are that univocal information is not available on the temporal stability of the saliva microbiome and on the influence of dental treatments; the risk of contamination with other microbiomes (for example, of the upper respiratory tract or bleeding gums) should be taken into consideration as well as that of contamination with environmental factors; population studies are lacking in order to establish useful frequencies in the forensic field; and further studies are needed on the statistical significance of the weight of this type of evidence in case we want to apply it to concrete cases.

The salivary microbiome is affected by various factors and seems to reflect the oral and general health status, with the bacterial composition associated with oral and systemic diseases [8]. Additionally, salivary microbial analysis provides information to identify an individual and/or his personal habits, age, ethnicity, and so on, providing useful clues in forensic contexts. Even if further studies are needed to relate the variation in the oral microbiome to specific factors, in order to understand how the salivary microbiome is influenced by an individual’s lifestyle, by reviewing the studies published so far, it is clear that the oral microbial analysis could become a useful forensic tool. Therefore, we believe that future studies should also focus on the collection of detailed information regarding the socio-cultural-economic status of volunteers, food habits, health status, and oral hygiene practices, instead of only being focused on the detection of microbiome variation related to one single factor, such as different geographic regions, age, relatedness, and a specific personal habit. These additional data could make it possible to infer, from the microbial analysis of a salivary trace collected from the crime scene, information that results in effectively narrowing the suspect pool. The lack of complete and wider information collection in experimental studies could compromise the possibility of gathering really informative data (i.e., useful to identify likely suspects in forensic contexts) from saliva microbiome analysis. For example, several studies published up till now have shown that salivary microbial composition can be related to the similar socio-economic background, culture, lifestyle, and food habits, which often characterize people coming from the same region [25,26,27,28]. In fact, factors such as food habits, which vary between different geographical locations, could contribute to the shaping of microbiome composition. For example, in the study conducted by Li et al. [27], the German group showed the greater alpha diversity. The authors attributed this result to the wider variety of food, with a great amount of different carbohydrates, which characterizes the German population. Nevertheless, recognizing the signature of ethnicity could become increasingly difficult owing to increasing globalization, with the adoption of new customs and habits by people living in places distant from their native region. Furthermore, such sensitive data may raise concerns for individual privacy if used for criminal investigations because of the fact that this technology allows clustering suspect populations that share common habits, customs, and traditions rather than identifying a single individual.

As already underlined in the field of skin microbial analysis, in order to gather high quality evidence that can really be utilized for indictment and conviction of the perpetrator of a crime, it is necessary to generate standards and guidelines that allow for the obtaining of statistically and scientifically meaningful results using the same methods under the same circumstances [35]. As recently pointed out by Neckovic et al. [36], the studies published so far about human microbial composition largely consist of descriptive, rather than functional, analyses of microbiomes.

Large-scale studies of the population of microbial communities in the saliva of hundreds of samples including several hundred individual sequences per sample are necessary to define oral microbial composition precisely. It is known that oral microbiota appears shortly after birth due to vertical transmission of bacteria from family members, especially from mother to child, but long-term surveys of a large number of subjects providing insight into the impact of different factors and the dynamics of microbiome changes are still lacking. Similar studies could allow us to understand whether all human beings share a “core” microbiome surrounded by a shell of diversity. It is also necessary to reach a more complete level of identification to create forensically motivated age/ethnicity/relatedness prediction models based on a microbiome’s characteristics, which are still lacking. Above all, given the fact that no standardized technique is available for the purpose of microbiome profiling, it is very difficult to collect comparable data from different studies; therefore, caution is required in interpreting the results and an effort to direct research towards studies that fill the current knowledge gaps is certainly useful.

Consequently, we believe that standardized systems should be developed and adopted worldwide for determining whether oral microbial signatures in a sample from a suspect match that in a sample from a crime scene. The next step will be to determine the probability that this match could have occurred by chance. The match probability calculation requires determining how commonly a particular microbial composition occurs. In fact, if the microbial pattern in the crime scene sample occurs commonly, the chance is higher that the microbial trace could have come from someone other than the suspect. Ideally, this probability would be determined by analysing the microbial composition of the entire population of people who could have plausibly left microbial traces at the crime scene and then calculating the frequency of the pattern of the microbial communities at the crime scene sample in this population. Obviously, because this approach is not concretely feasible, it is important to build reference databases categorized by, for example, age, race, habits, and ethnicity, to calculate probabilities and to perform deep sequencing in order to detect bacterial genera that have not been previously reported in databases.

To ensure that the analytical methodology is accurate, specific, and reproducible, validation processes should also be conducted. The accurate interpretation of the obtained results depends on the development of bio-informatic processing and data analysis and the implementation of updated microbial databases.

Therefore, several studies are still needed to allow the microbial salivary trace evidence to be used in a court of law to prosecute an individual who has been arrested while achieving the standards for admissibility of scientific evidence in a legal setting.

## 5. Conclusions

In conclusion, we consider the establishment of a salivary “microbial fingerprint” from crime scene traces as a possible approach to address various forensically relevant questions that cannot be answered through classical forensic genetics. Oral microbiome analysis could provide some kind of evidence in investigating criminal cases, linking criminals to a crime scene. The journey to transform forensic microbiology into a standard and widely accepted forensic tool for personal identification is still long and difficult because of all the limitations that have been recently identified. Nevertheless, the studies conducted so far have unequivocally shown an enormous forensic potential to be developed. Therefore, it would be a shame to miss this opportunity and give up, and it is worthwhile to implement this area of research to prevent a lack of knowledge or technical difficulties from frightening us and preventing us from going beyond our limits.

## Figures and Tables

**Table 1:** Potentials and limitations for forensic purposes of oral microbiome, considering the four categories: (a) personal identification for forensic purposes; (b) oral microbiome in age prediction; (c) signature of ethnicity in oral microbiome; (d) oral microbiome as indicator of personal habits: smoking.

Saliva Microbiome in Forensics	
Personal identification for forensic purposes	
Rasiah et al., 2005 [15] Costello et al., 2009 [16] Lazarevic et al., 2010 [17] Zhu et al., 2012 [18] Stahringer et al., 2010 [19] Leake et al., 2016 [13] Wang et al., 2019 [22] Sundström et al., 2020 [23]	**Suitability for forensic purposes:** As the salivary microbial composition seems to undergo little temporal changes, resembling itself even after temporal interval of years, showing a long-term stability, the possibility to differentiate individuals by analysing their microbial signature appears promising in forensics to link crime scene’s traces to the subject(s) who left them. Methods based on the analysis of inter-individual variation of the salivary microbiome could allow human identification especially when current methods, based on human DNA typing, cannot be utilized. **Limits:** The lack of standardized systems makes difficult the accurate interpretation of the obtained results. Further investigations should be performed to clearly determine the stability of such samples and specificity/sensitivity of such analyses in the context of forensics.
**Oral microbiome in age prediction**	
Huang et al., 2020 [24] Murugesan et al., 2020 [25]	**Suitability for forensic purposes:** The age prediction of an unknown trace donor could allow the narrowing of the suspects’ pool. Salivary microbial analysis could pinpoint a subject’s age within 5 years and a low bacterial richness could indicate that the trace belongs to an individual aged more than 65 years. **Limits:** It is difficult to establish if the changes in oral microbiome related to aging so far highlighted are linked to health conditions.
**Signature of ethnicity in oral microbiome**	
Nasidze et al., 2009 [26] Li et al., 2014 [27] Sarkar et al., 2017 [28] Murugesan et al., 2020 [25]	**Suitability for forensic purposes:** The studies focusing on the effect caused by different geographic and climatic areas on the salivary microbial communities have highlighted that it is possible to infer a signature of ethnicity by analysing oral microbial composition, in order to guide police investigations towards the most likely group of potential suspects. **Limits:** The adoption of new customs and habits by people living in places distant from the native region could make it difficult to recognize an “original” microbiome signature, mostly linked to the native ethnic group. Clustering suspect populations that share common habits, customs, and traditions rather than identifying a single individual may raise privacy concerns and could lead to discrimination and stigmatization.
**Oral microbiome as indicator of personal habits: smoking**	
Kato et al., 2016 [29] Wu et al., 2016 [31] Yu et al., 2017 [30] Yang et al., 2019 [32] Grine et al., 2019 [33] Al-Zyoud et al., 2020 [34] Murugesan et al., 2020 [25]	**Suitability for forensic purposes:** The microbial diversity and composition seem to be significantly affected by smoking status on the buccal mucosa. Tobacco smoking seems to reduce the diversity of saliva microbial composition. **Limits:** Smoking-related changes are not permanent and the bacteria depleted by cigarette smoking could be restored after quitting smoking. At the moment, evidence related to the impact of cigarette smoking on the oral microbiome does not seem to be promising as a marker for microbiome signature.
